# Supplementary material for: Streamlining effects of extra telomeric repeat on telomeric DNA folding revealed by fluorescence-force spectroscopy
Source: Nucleic Acids Res. 2019 Oct 16;47(21):11044–56. doi: 10.1093/nar/gkz906 (PMC6868435; doi:10.1093/nar/gkz906)
Supplement: gkz906_Supplemental_File [file gkz906_supplemental_file.pdf]

Supporting Information

Streamlining effects of extra telomeric repeat on telomere folding revealed by fluorescence-force spectroscopy

Jaba Mitra[1,2] *and Taekjip Ha*[2,3,4,5*]

[1]*Department of Materials Science and Engineering, University of Illinois at Urbana-Champaign, Urbana IL, USA;* [2]*Department of Biophysics and Biophysical Chemistry, Johns Hopkins University, Baltimore, MD, USA;* [3]*Department of Biophysics, Johns Hopkins University, Baltimore, MD, USA;* [4]*Department of Biomedical Engineering, Johns Hopkins University, Baltimore, MD, USA;* [5]*Howard Hughes Medical Institute, USA*

*To whom correspondence should be addressed: tjha@jhu.edu

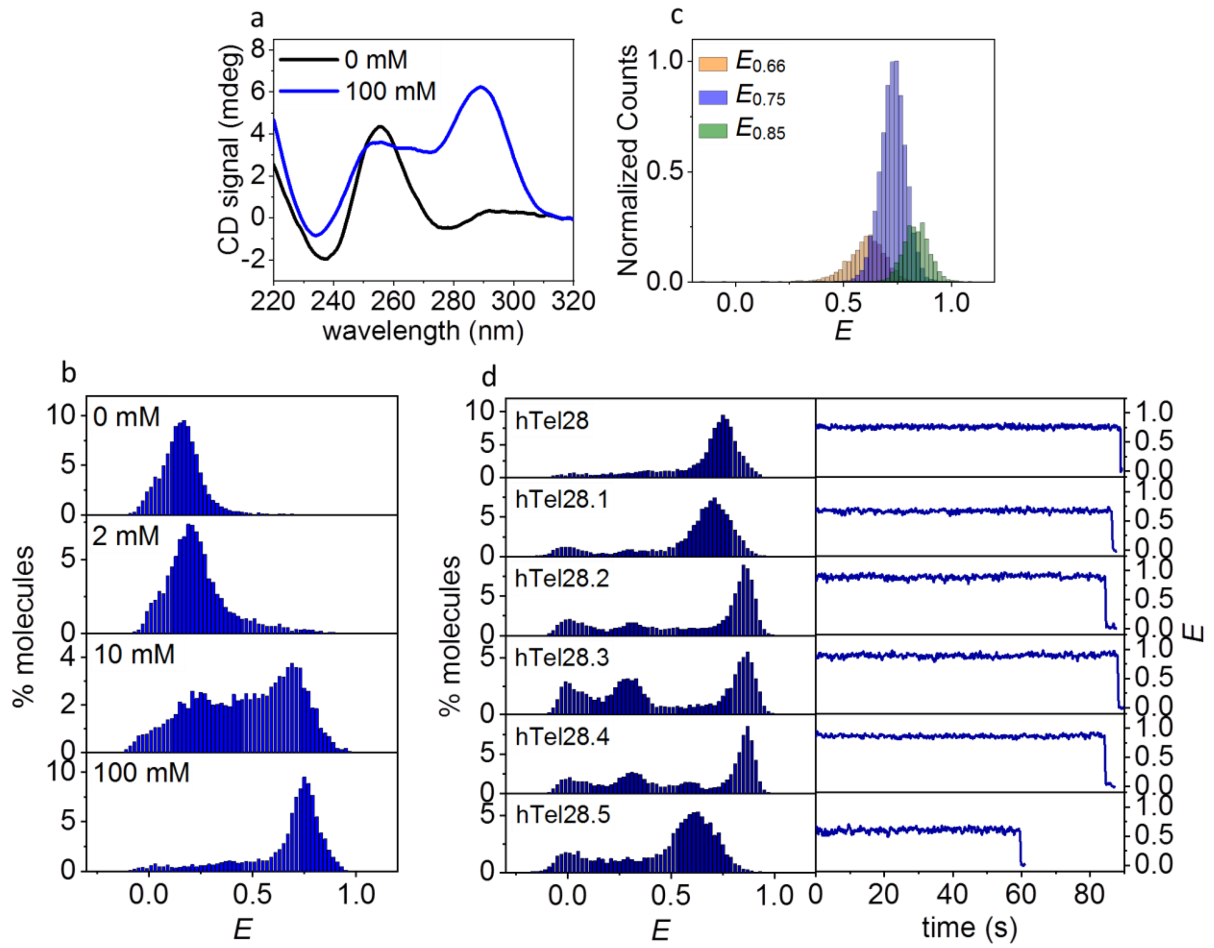

Fig. S1: (a) CD spectra of hTel28 in 0 and 100 mM K^+. (b) *E* histograms of hTel28 as a function of K^+ concentration. (c) *E* histograms recast from time trajectories of hTel28 in 100 mM K^+, at *E* values of ~ 0.68, 0.75 and 0.86. The single molecule time trajectories were categorized based on the average *E* values and the *E* histograms were plotted by averaging the first ten time points from each trajectory. (d) *E* histograms (left) and single molecule time trajectories (30 ms integration time, right) of hTel28 mutants in 100 mM K^+ in the absence of force.

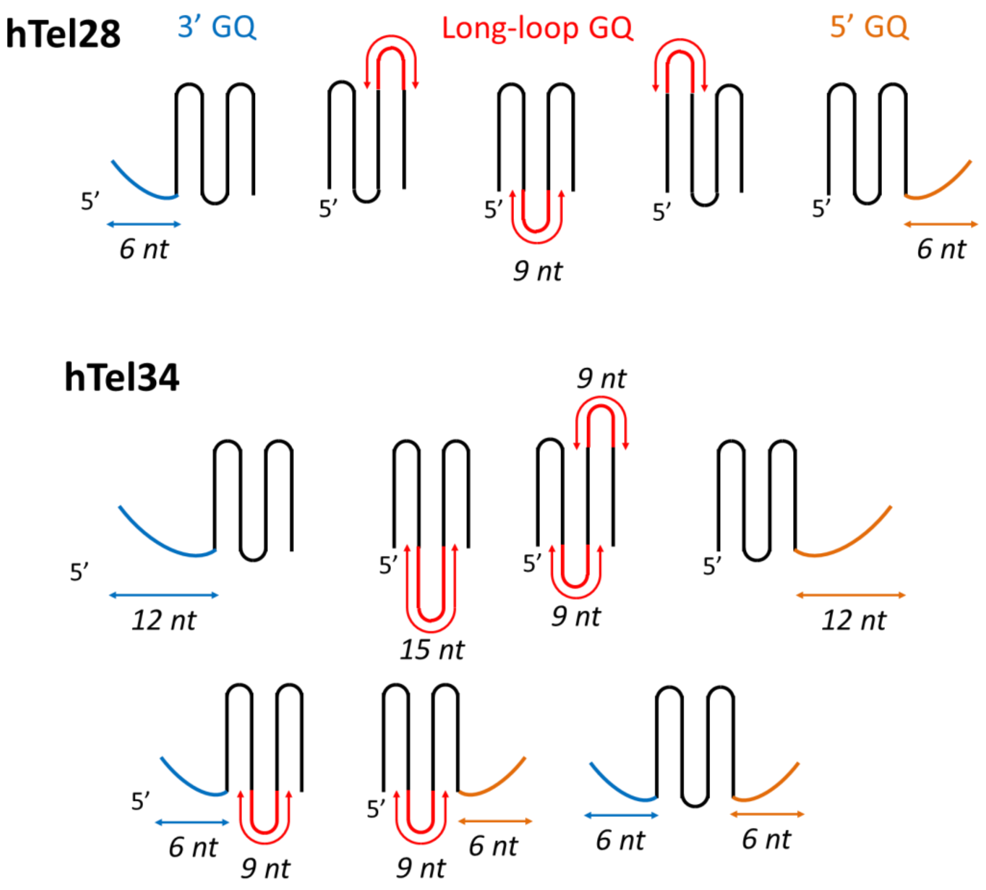

Fig. S2: GQ formation in hTel28 and hTel34. In hTel28, GQs can form at the termini i.e. 3' or 5' end (hTel28.1 and hTel28.5 respectively) or by association of a 9 nt long loop bearing a T_2AG_3 segment (e.g. hTel28.2, hTel28.3 or hTel28.4). The unassociated T_2AG_3 segment at the 5' and 3' ends are shown in blue and orange respectively whereas the long loop is shown in red. The conformations represent hTel28.1 to hTel28.5 from left to right.

In hTel34, GQ can form at the 3' or 5' end with a 12 nt long ssDNA tail (two conformations differing in GQ polarity). Long-loop GQs can harbor a 15 nt long loop harboring two T_2AG_3 segments (3 conformations), two 9 nt long loops with a T_2AG_3 segment each (3 conformations) or a 9 nt loop with a ssDNA tail at the 5' or 3' end (6 conformations). Additionally, GQ formation may involve two 6 nt overhangs at both the 3' and 5' ends (1 conformation). Fifteen conformations in all can be conceived based on the above prototypes.

The conformations illustrated only take into account the differences in the relative position of the unpaired T_2AG_3 segment, irrespective of the structure of the GQ formed.

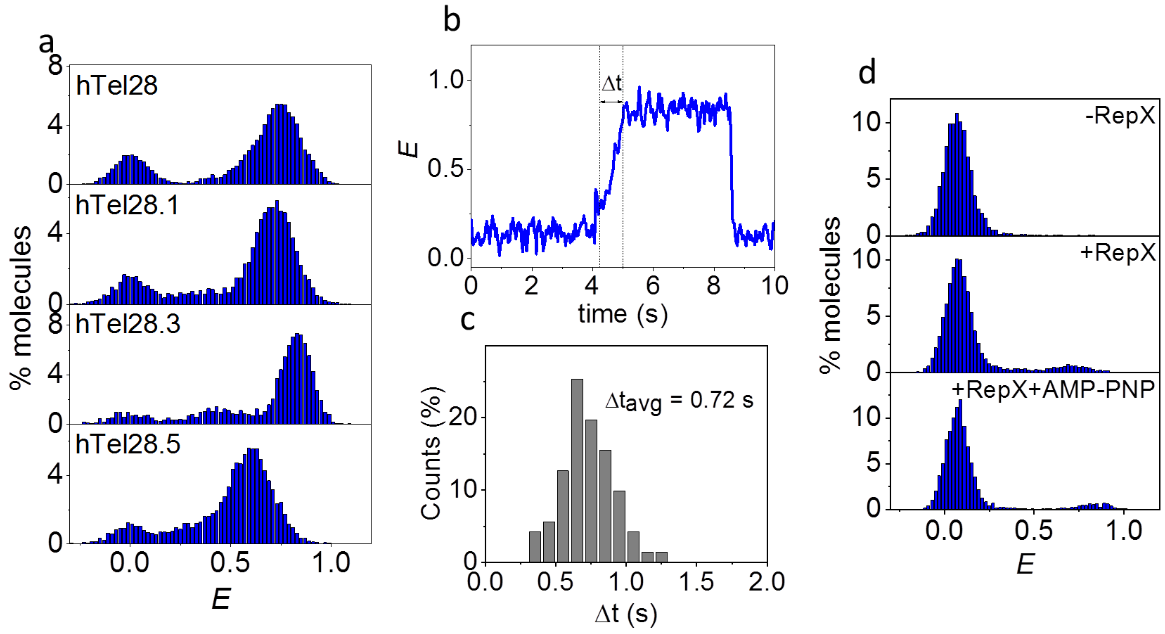

Fig. S3: (a) *E* histograms of hTel28, hTel28.1, hTel28.3 and hTel28.5 in the Rep-X unwinding buffer. (b) Real-time *E* trajectory of unwinding of hTel28-duplex and folding of GQ in Δt time (30 ms integration time). (c) Histogram of Δt. The cumulative time for unwinding of the hTel28-duplex and 3'-GQ folding is estimated as 0.72±0.18 s. (d) *E* histograms of hTel28-duplex in the Rep-X unbound (top) and bound (middle) states, and after Rep-X unwinding of the duplex in presence of 1 mM AMP-PNP and 2.5 mM $MgCl_2$ (bottom).

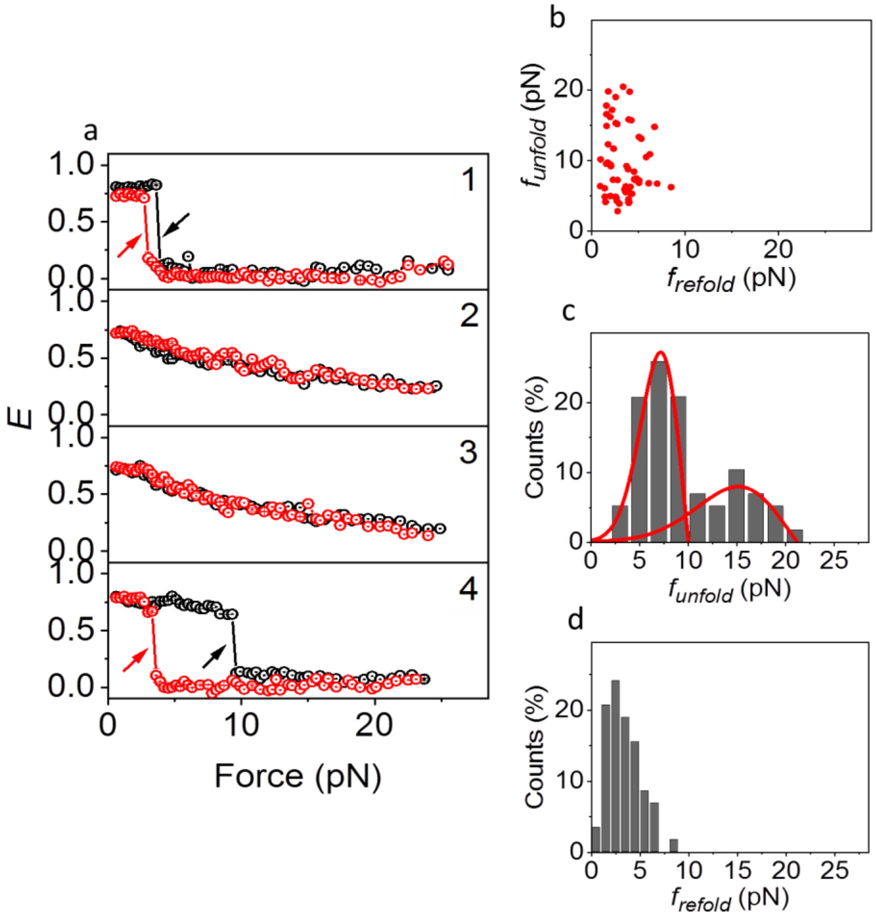

Fig. S4: (a) E vs force response of the hTel28 molecule shown in Fig. 3b. The pulling cycle # is depicted in the inset. Stretching and relaxation are represented in black and red respectively. Cycles 1 and 4 show complete unfolding, whereas cycles 2 and 3 show partial (I) unfolding. The arrows denote f_{unfold} (black) and f_{refold} (red). (b) A scatter plot of f_{unfold} and f_{refold} of complete unfolding events (N=58). (c and d) f_{unfold} (c) and f_{refold} (d) histograms corresponding to complete unfolding events (N=58). The red curves represent the rupture force distributions predicted using the Dudko-Szabo model (1,2). All measurements were done in 100 mM K^+.

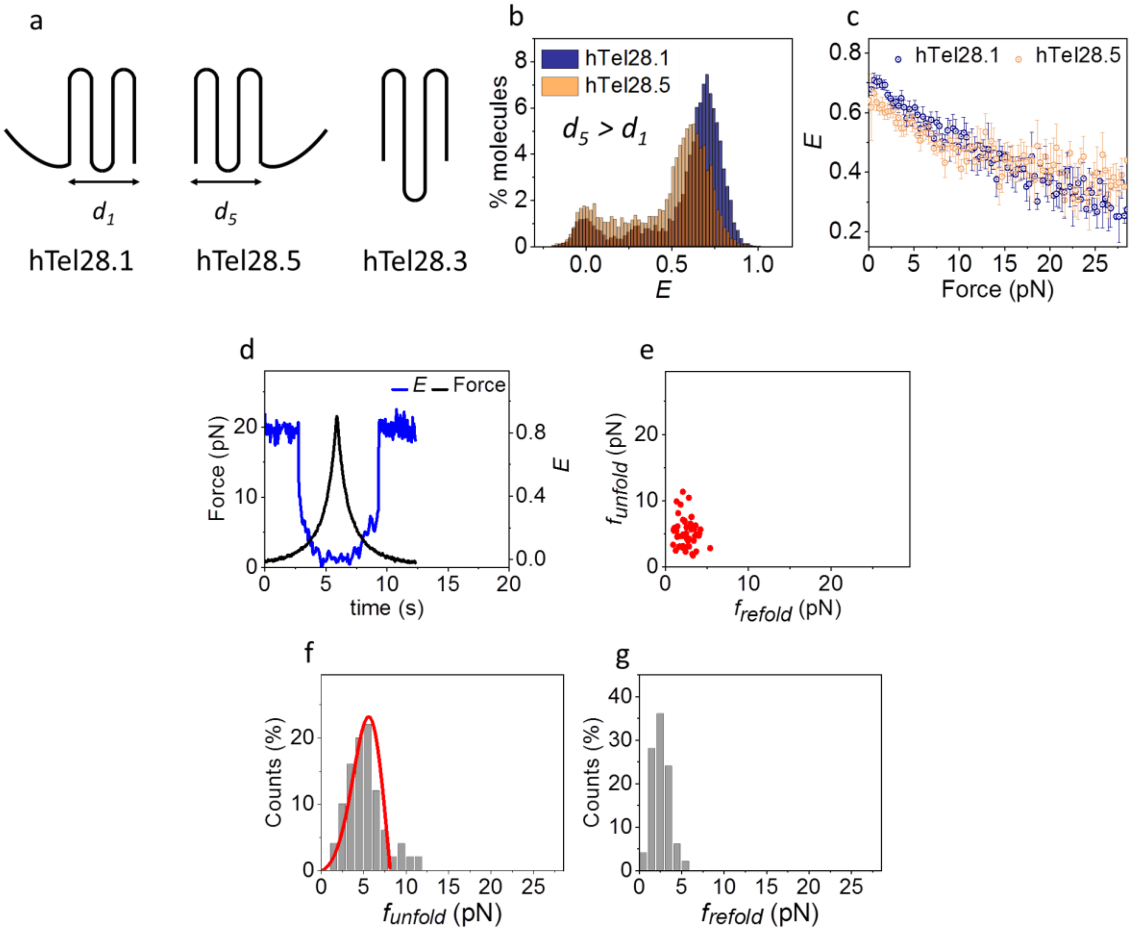

Fig. S5: (a) A cartoon depicting polarized GQ formation at the 3' (hTel28.1) and 5' (hTel28.5) end or via association of a long loop (hTel28.3). The end to end distances of GQs formed in hTel28.1 and hTel28.5 (excluding the ssDNA tail), are represented by d_1 and d_5 respectively. (b) Overlap of *E* histograms of hTel28.1 and hTel28.5 in 100 mM K^+. (c) Overlap of average *E* vs force response (stretch) of hTel28.1 and hTel28.5 in 100 mM K^+. (d) A representative *E* time trace of hTel28.3 in one pulling cycle (20 ms integration time). (e) A scatter plot of f_{unfold} and f_{refold} (*N*=50). (f and g) f_{unfold} (f) and f_{refold} (g) histograms corresponding to complete unfolding events (*N*=50). The red curve represents the rupture force distribution predicted using the Dudko-Szabo model (1,2). All measurements were done in 100 mM K^+.

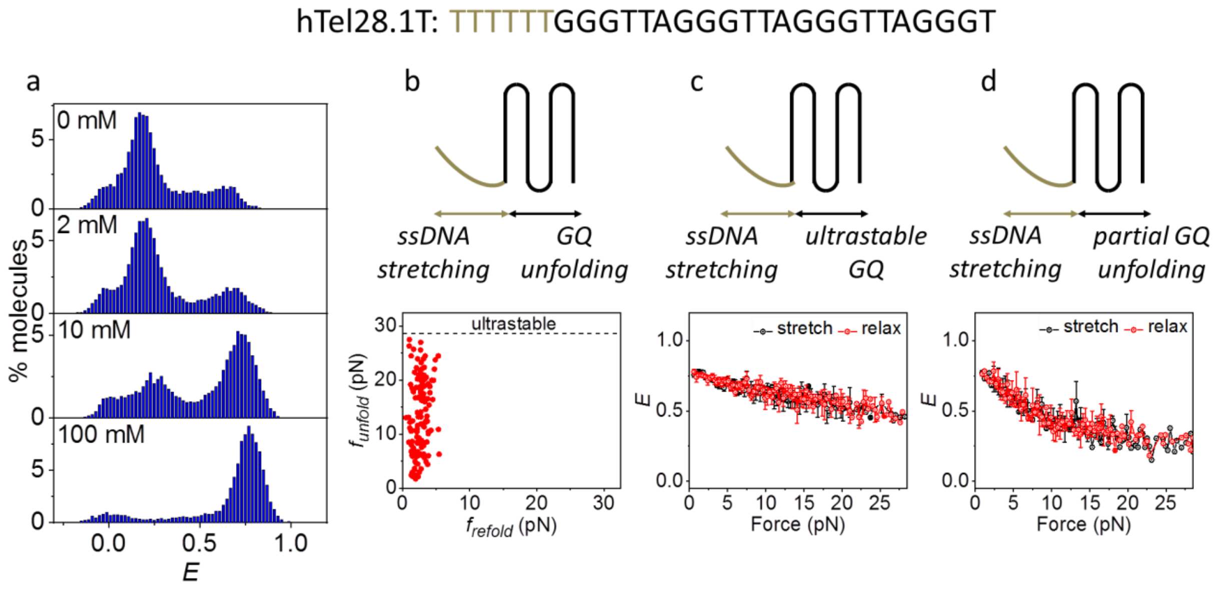

Fig. S6: (a) E histograms of hTel28.T as a function of K^+ concentration. (b-d) Schematic depicting three different mechanical responses of hTel28.1T in 100 mM K^+. A scatter plot of f_{unfold} and f_{refold} is shown in (b, lower panel, N=131). Average E vs force responses of ultrastable and partially unfolded GQs are shown in (c, N=84) and (d, N=24).

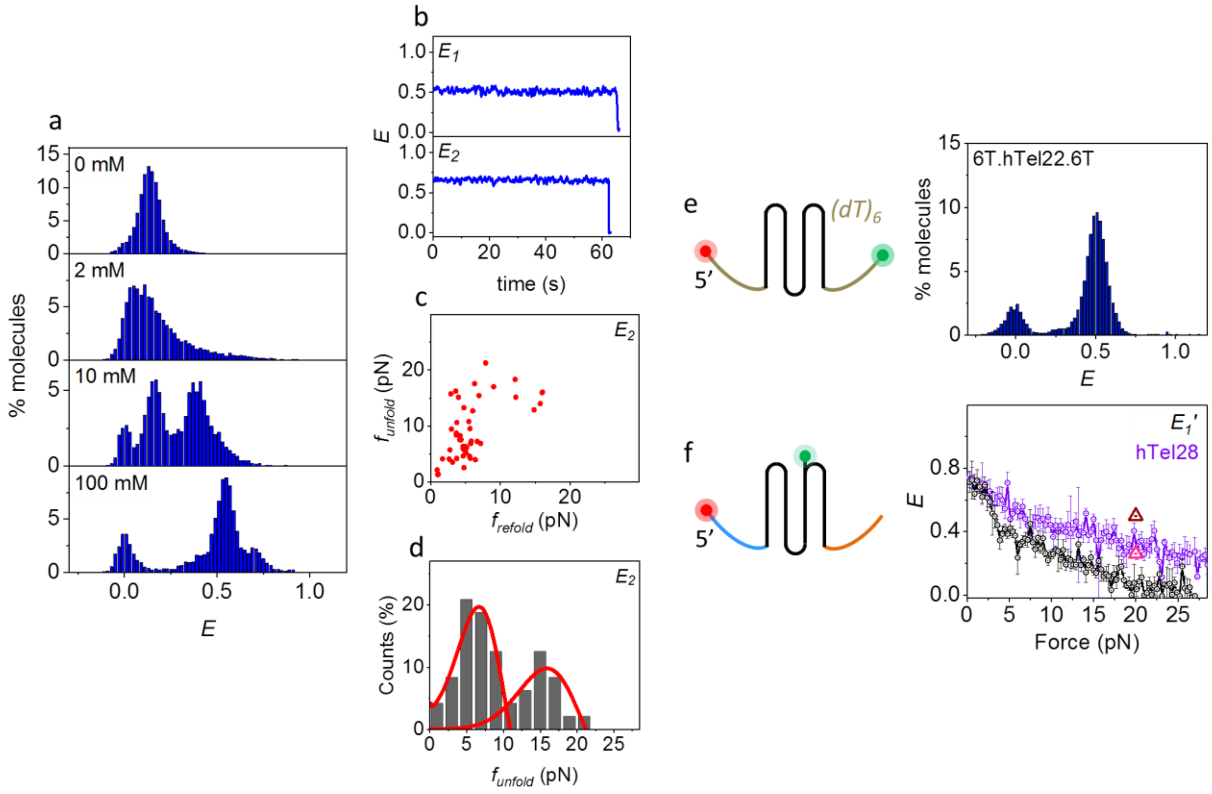

Fig. S7: (a) E histogram of hTel34 as a function of K^+ concentration in the absence of force. (b) Representative E time traces of E_1 and E_2 (30 ms integration time) in absence of force. (c) A scatter plot of f_{unfold} and f_{refold} of E_2. (d) Histogram of f_{unfold}. The red curves represent the rupture force distributions

predicted using the Dudko-Szabo model (1,2). N=48 for (c) and (d). (e) Schematic representation (left) and E histogram (right) of 6T.hTel22.6T in 100 mM K^+. (f) Schematic representation of hTel34' in reference to (e) (left). As the GQ core is inherently highly compact, hTel34' topology would resemble hTel28 (or, hTel28.1T). Comparison of E vs force curves (stretch) of E_1' and hTel28 (f, right). Computed FRET values at 20 pN corresponsing to strteching of 6 nt (brown triangle) and 12 nt (magenta triangle) only (3), while the GQ core remains unperturbed.

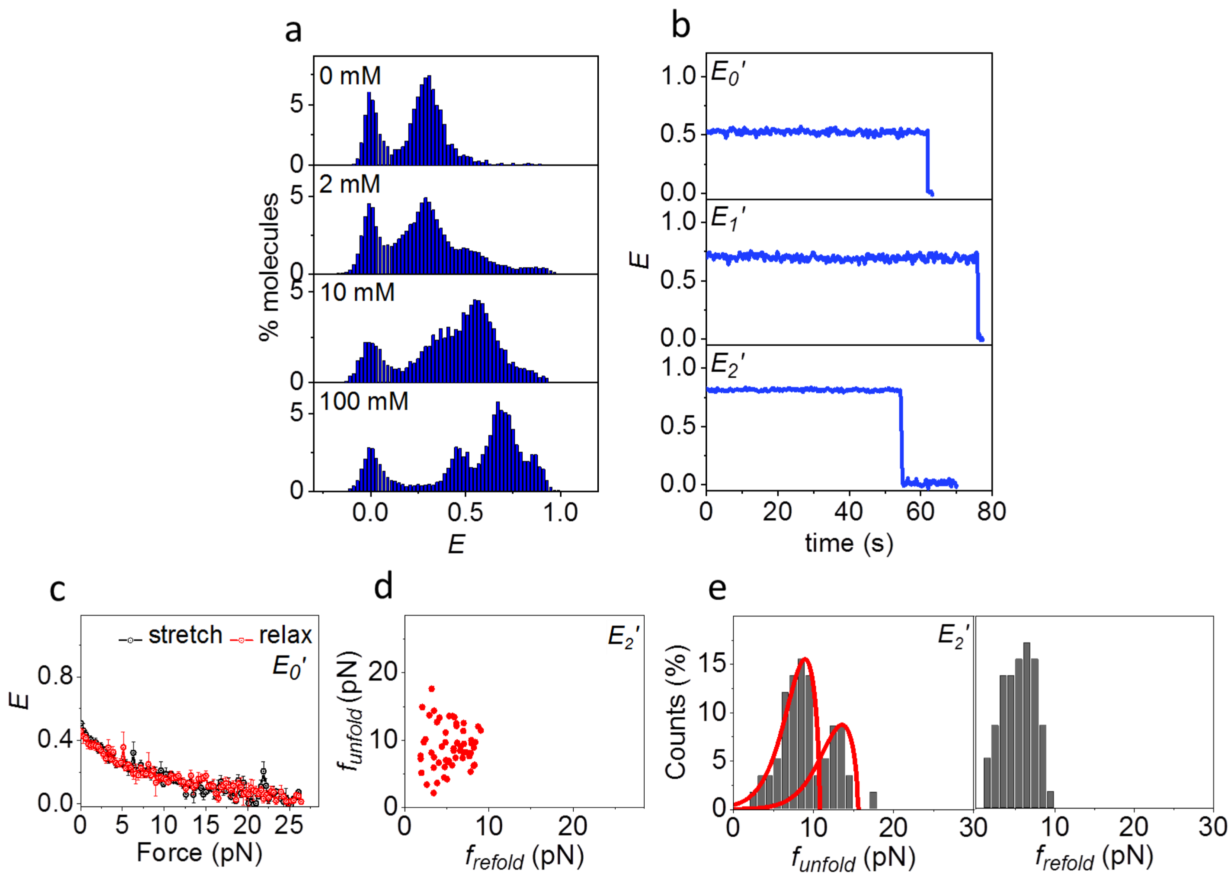

Fig. S8: (a) E histogram of hTel34' as a function of K^+ concentration in the absence of force. (b) Representative E time traces of E_0', E_1' and E_2' in 100 mM K^+ in absence of force (30 ms integration time). (c) Average E vs force response of E_0'. (d) A scatter plot of f_{unfold} and f_{refold} of E_1'. (e) Histograms of f_{unfold} and f_{refold} for E_1'. The red curves represent the rupture force distributions predicted using the Dudko-Szabo model (1,2). N=58 for (d) and (e).

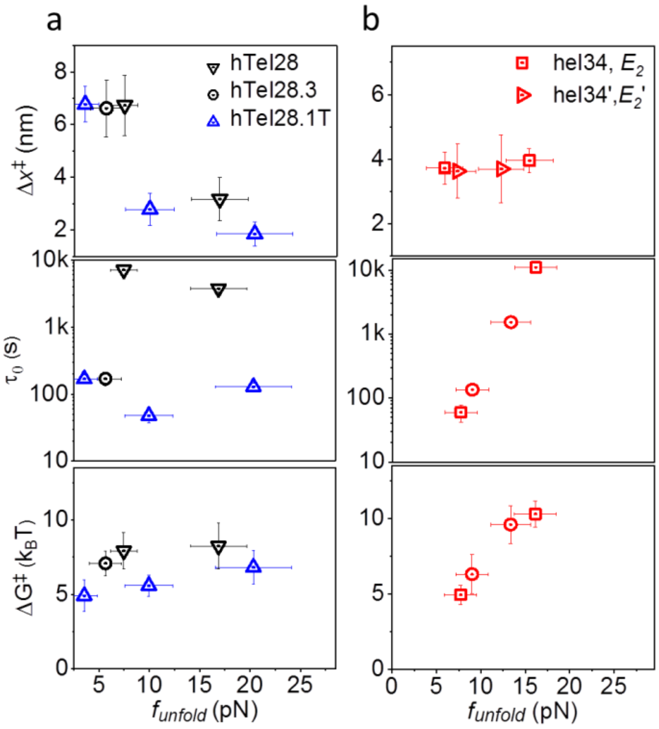

Fig. S9: $\Delta x^{\ddagger}$s (top), $\tau_u(0)$ (middle) and $\Delta G^{\ddagger}$s (bottom) corresponding to the force clusters observed in hTel28, hTel28.3 and hTel28.1T (a) and hTel34 and hTel34' (b) in 100 mM K^+.

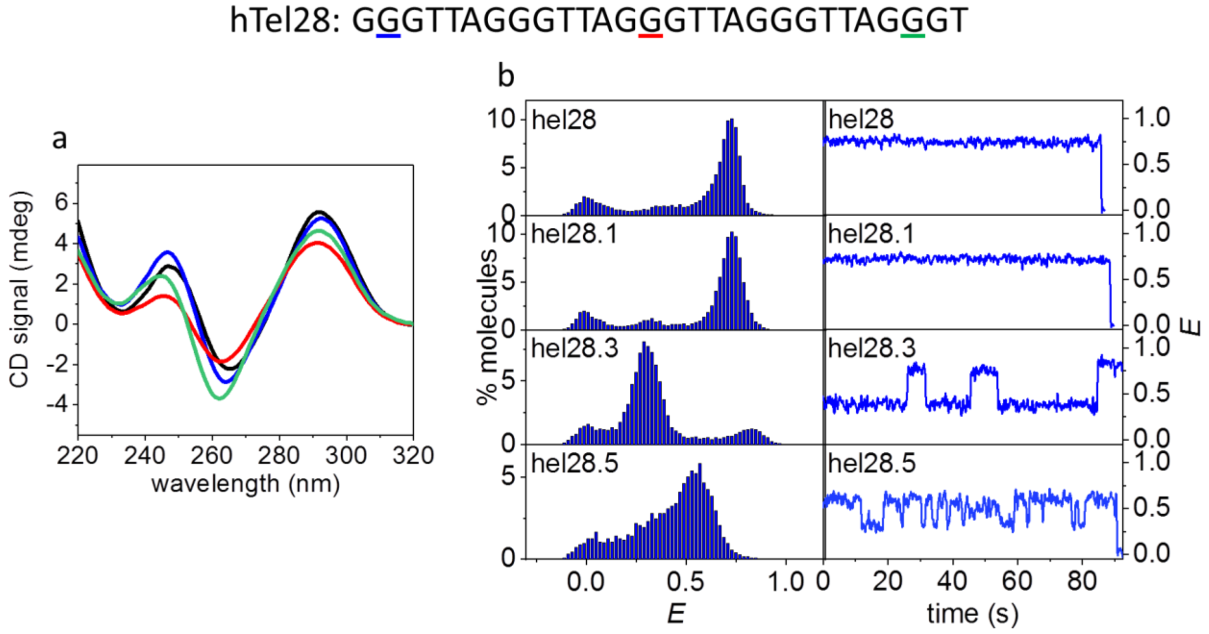

Fig. S10: (a) CD spectra of hTel28 (blue), hTel28.1 (navy blue), hTel28.3 (red) and hTel28.5 (green) in 100 mM Na^+. The G to T mutation sites in hTel28 corresponding to hTel28.1, hTel28.3 and hTel28.5 are underlined on the sequence with navy blue, red and green respectively. (b) E histograms (left) and

representative time traces (right, 30 ms integration time) of hTel28 variants in 100 mM Na^+ in absence of force.

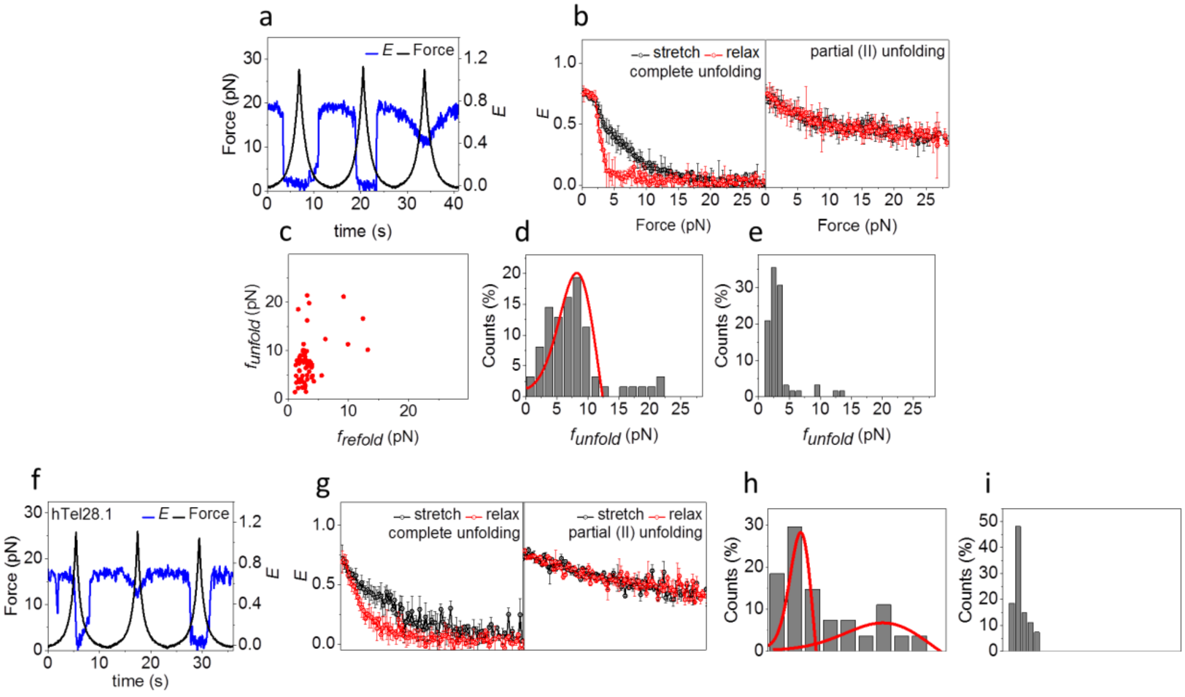

Fig. S11: (a) A representative *E* time trace of hTel28 in three pulling cycles. (b) Average *E* vs force response of molecule showing complete (*N*=73, left) and partial (II) (*N*=31, right) unfolding. (c) A scatter plot of f_{unfold} and f_{refold} corresponding to (b, left) (*N*=73). (d and e) Histograms of f_{unfold} (d) and f_{refold} (e) corresponding to complete unfolding events only. (f) Representative *E* time trace of hTel28.1. (g) Average *E* vs force response of hTel28.1. (h and i) Histograms of f_{unfold} (h) hTel28.1 and f_{refold} (i) of hTel28.1 showing complete unfolding events only. (*N*=27 for hTel28.1). All measurements were done in 100 mM Na^+, at an integration time of 20 ms.

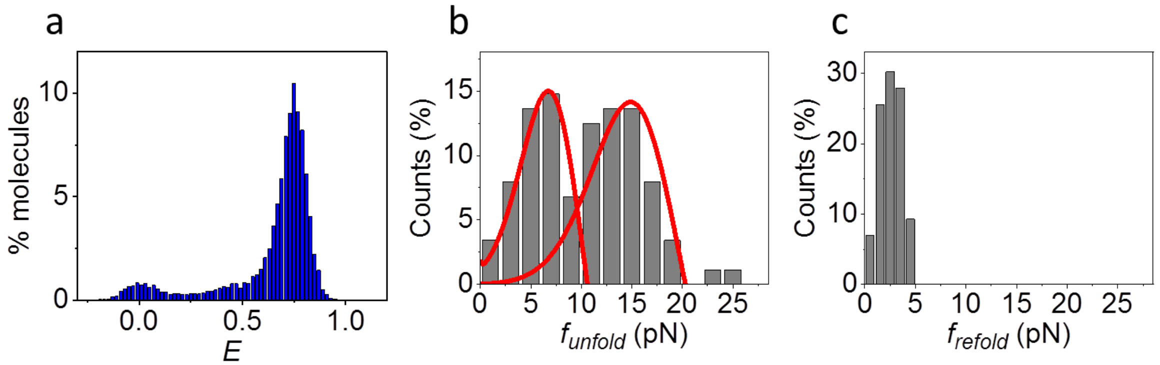

Fig. S12: (a) *E* histogram of hTel28.1T in 100 mM Na^+, in the absence of force. (b and c) Distributions of f_{unfold} (b) and f_{refold} (c) (*N*=88). The red curves represent the rupture force distributions predicted using the Dudko-Szabo model (1,2).

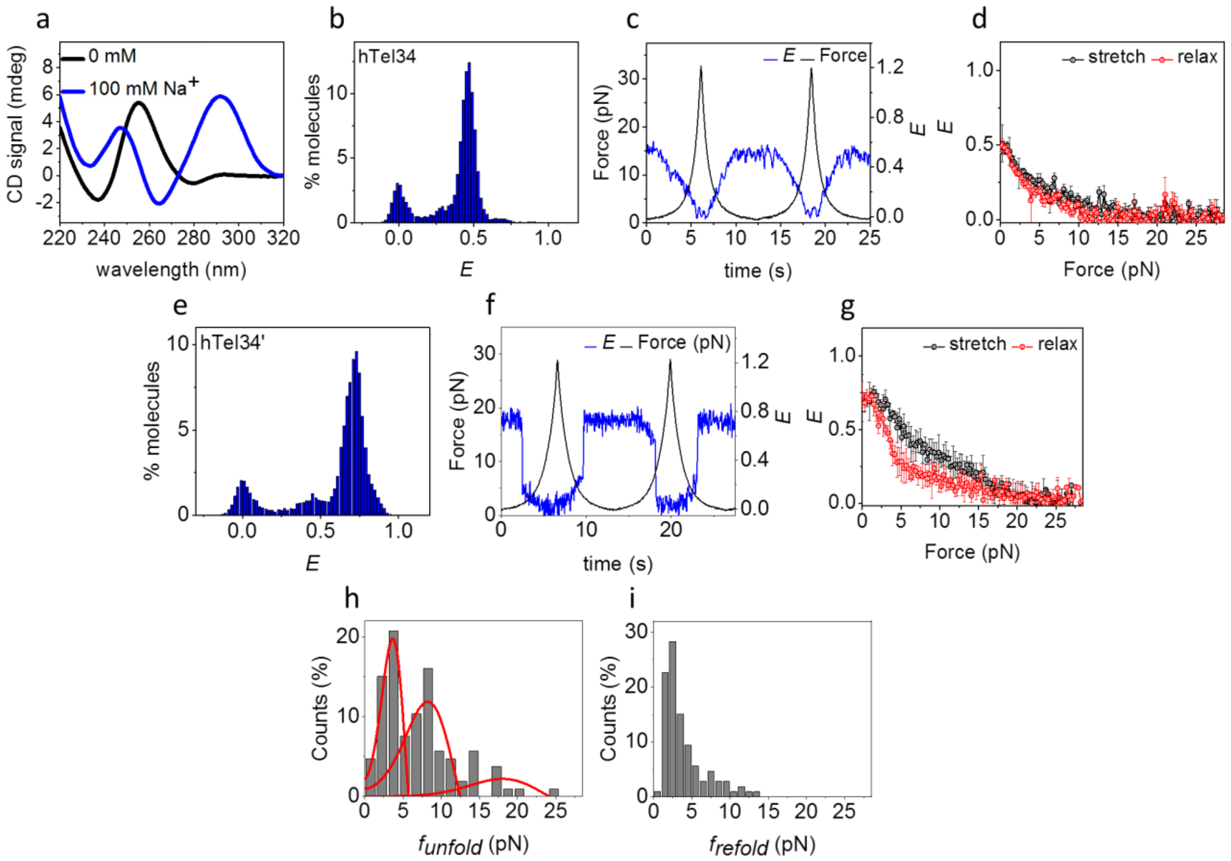

Fig. S13: (a) CD spectra of hTel34. (b) *E* histogram of hTel34 in the absence of force (c) A representative *E* time trace showing two pulling cycles. (d) Average *E* vs force response of hTel34. (e) *E* histogram of hTel34'. (f) A representative *E* time trace showing three pulling cycles. (g) Average *E* vs force response of hTel34'. (h and i) Histograms of f_{unfold} (h) and f_{refold} (i) (N=105). The red curves represent the rupture force distributions predicted using the Dudko-Szabo model (1,2). The data in (b-i) were collected in 100 mM Na^+. (c) and (f) were collected at an integration time of 20 ms.

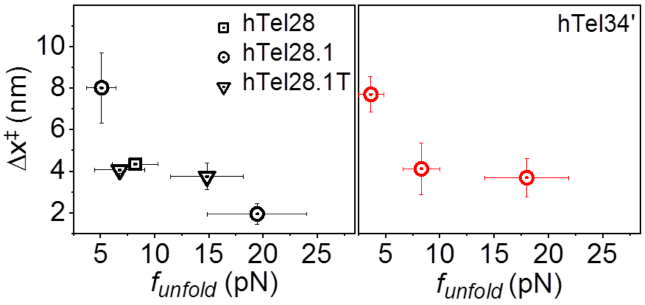

Fig. S14: $\Delta x^{\ddagger}$s corresponding to the force clusters observed in hTel28, hTel28.1, hTel28.5, hTel28.1T and hTel34' in 100 mM Na^+.

All error bars represent standard errors.

References

1. Dudko, O.K., Hummer, G. and Szabo, A. (2006) Intrinsic rates and activation free energies from single-molecule pulling experiments. *Phys Rev Lett.*, **96**, 108101.
2. Dudko, O.K., Hummer, G. and Szabo, A. (2008) Theory, analysis, and interpretation of single-molecule force spectroscopy experiments. *Proc. Natl. Acad. Sci. U S A.*, **105**, 15755-15760.
3. Maffeo, C., Ngo, T.T.M., Ha, T. and Aksimentiev, A. (2014) A Coarse-Grained Model of Unstructured Single-Stranded DNA Derived from Atomistic Simulation and Single-Molecule Experiment. *J. Chem. Theory Comput.*, **10**, 2891-2896.
